# Patent ductus arteriosus, tracheal ventilation, and the risk of bronchopulmonary dysplasia

**DOI:** 10.1038/s41390-021-01475-w

**Published:** 2021-03-31

**Authors:** Ronald I. Clyman, Nancy K. Hills, Gilles Cambonie, Thierry Debillon, Isabelle Ligi, Geraldine Gascoin, Juliana Patkai, Alain Beuchee, Geraldine Favrais, Xavier Durrmeyer, Cyril Flamant, Jean Christophe Rozé

**Affiliations:** 1 Department of Pediatrics and Cardiovascular Research Institute, University of California San Francisco, San Francisco, CA USA; 2 Departments of Epidemiology and Biostatistics, and Neurology, University of California San Francisco, San Francisco, CA USA; 3 Neonatal Medicine, Montpellier University Hospital, Montpellier, France; 4 Department of Neonatalogy, University Hospital of Grenoble, Grenoble, France; 5 Department of Neonatalogy, Assitance Publique Hôpitaux de Marseille, Marseille, France; 6 Neonatal Medicine, Angers University Hospital, Angers, France; 7 Neonatal Intensive Care Unit, Cochin Hospital Maternity of Port-Royal, Paris, France; 8 Department of Neonatalogy, Rennes University Hospital, Rennes, France; 9 Department of Neonatalogy, Tours University Hospital, Tours, France; 10 Department of Neonatalogy, Centre Hospitalier Intercommunal de Créteil, Créteil, France; 11 Faculté de Médecine de Créteil, Université Paris Est Créteil, IMRB, GRC CARMAS, Créteil, France; 12 Department of Neonatalogy, Nantes University Hospital, Nantes, France; 13 Centre d’Investigation Clinique CIC1413, INSERM-Nantes University Hospital, Nantes, France

## Abstract

**Background:**

An increased risk for bronchopulmonary dysplasia (BPD) exists when moderate-to-large patent ductus arteriosus shunts (hsPDA) persist beyond 14 days.

**Goal:**

To examine the interaction between prolonged exposures to tracheal ventilation (≥10 days) and hsPDA on the incidence of BPD in infants <28 weeks gestation.

**Study Design:**

Predefined definitions of prolonged ventilation (≥10 days), hsPDA (≥14 days), and BPD (room air challenge test at 36 weeks) were used to analyze deidentified data from the multicenter TRIOCAPI RCT in a secondary analysis of the trial.

**Results:**

Among 307 infants who survived >14 days, 41 died before 36 weeks. Among survivors, 93/266 had BPD. The association between BPD and hsPDA depended on the length of intubation. In multivariable analyses, prolonged hsPDA shunts were associated with increased BPD (odds ratio (OR) (95% confidence interval (CI)) = 3.00 (1.58–5.71)) when infants required intubation for ≥10 days. In contrast, there was no significant association between hsPDA exposure and BPD when infants were intubated <10 days (OR (95% CI) = 1.49 (0.98–2.26)). A similar relationship between prolonged hsPDA and length of intubation was found for BPD/death (*n* = 307): infants intubated ≥10 days: OR (95% CI) = 2.41 (1.47–3.95)); infants intubated <10 days: OR (95% CI) = 1.37 (0.86–2.19)).

**Conclusions:**

Moderate-to-large PDAs were associated with increased risks of BPD and BPD/death—but only when infants required intubation ≥10 days.

**Impact:**

- Infants with a moderate-to-large hsPDA that persist beyond 14 days are only at risk for developing BPD if they also receive prolonged tracheal ventilation for ≥10 days.
- Infants who receive less ventilatory support (intubation for <10 days) have the same incidence of BPD whether the ductus closes shortly after birth or whether it persists as a moderate-to-large shunt for several weeks.
- Early PDA closure may be unnecessary in infants who require short durations of intubation since the PDA does not seem to alter the incidence of BPD in infants who require intubation for <10 days.

## Introduction

Between 50 and 70% of infants <28 weeks gestation have a patent ductus arteriosus (PDA) that persists for weeks after birth.^1^ The left-to-right PDA shunt increases the risk of several neonatal morbidities (dopamine-dependent hypotension, early hemorrhagic pulmonary edema, and the intensity of respiratory support) that occur during the first week after birth, and early PDA closure can decrease the incidence of these morbidities.^2–6^

Whether exposure to a PDA shunt increases the risks of later neonatal morbidities, like bronchopulmonary dysplasia (BPD), is still unclear. Preclinical trials, in preterm baboons that required intubated tracheal ventilation for 14 days, found that exposure to a moderate-to-large PDA shunt for 2 weeks caused an arrest in alveolar development (the hallmark of BPD).^7^ On the other hand, none of the human randomized clinical trials (RCTs) performed to date have found a relationship between therapies intended to close the PDA and the risk of developing BPD.^5,8–13^ Unfortunately, when the human clinical trials were initially designed there was little information available to determine which infants with a PDA were actually at risk for developing BPD (i.e., which infants might actually benefit from being enrolled in such a treatment trial). As a result, little attention was paid to either the magnitude of the PDA shunt, the duration of shunt exposure, or the infant’s need for respiratory support. This is an important concern when trying to interpret the trials’ results.

Several recent single-center observational studies have shown that infants with small PDA shunts do not appear to be at increased risk for developing BPD nor are infants exposed to moderate-to-large shunts for <7–10 days. In these observational studies, the association between PDA and BPD only occurred when moderate-to-large shunts were present and persisted beyond 10–14 days.^14–18^

An infant’s need for invasive respiratory support also may play an important role in determining whether prolonged PDA exposure is associated with BPD. Two recent preliminary studies, from nurseries primarily based in the United States, found an interaction between the duration of tracheal ventilation and the duration of PDA exposure such that the effect of prolonged PDA exposure on BPD differed depending on whether the baby required tracheal ventilation for brief or prolonged periods.^18,19^ In these studies, the association between a moderate-to-large PDA and BPD was only observed among infants who required intubated tracheal ventilation for ≥10 days;^18,19^ the incidence of BPD among infants who required intubation for shorter durations (<10 days) was the same whether the ductus closed soon after birth or whether it persisted as a moderate-to-large shunt for several weeks.^18,19^ The results of these preliminary studies need to be replicated in other centers before they can be thought of as predictable and consistent criteria for identifying infants at increased risk for developing BPD in the presence of a moderate-to-large PDA. Therefore, we used data from the recently completed TRIOCAPI (TRaitement CIblé et PrécOce du Canal Artériel du Prématuré par Ibuprofène) trial to confirm their findings.

The TRIOCAPI trial (NCT01630278) (a double-blind, multicenter, randomized, placebo-controlled, trial conducted between 2012 and 2017 at 11 French tertiary-care neonatal units^20^) enrolled infants <28 weeks gestation to determine if routine early ultrasound-guided pharmacologic treatment of moderate-to-large patent ductus arteriosus would increase survival without cerebral palsy. We performed a secondary analysis of the trial’s deidentified data to determine whether the association between PDA exposure and BPD differed depending on the length of time that infants required tracheal ventilation and to confirm the previous findings. We hypothesized that moderate-to-large PDAs would be associated with an increased incidence of BPD in infants who received prolonged tracheal ventilation (≥10 days), but not in those who received less ventilatory support (intubation for <10 days).

## Methods

The goal of our study was to validate the findings of the two prior studies^18,19^ that found an interaction between the duration of tracheal ventilation and the duration of PDA exposure on the incidence of BPD. As a validation study, we used the same definitions for our study variables and outcomes that were used in the prior studies (see below for definitions).

We utilized deidentified data from the multicenter TRIOCAPI trial (NCT01630278).^20^ Approvals by the national ethics committee (Comite de Protection des Personnes Ouest IV), the French National Drug Safety Agency (ANSM, EudraCT number 2011-003063-3), and the French data protection authority (Commission Nationale de l’Informatique et des Libertes) and written informed parental consent were obtained before patient enrollment into the trial.

The parent TRIOCAPI trial enrolled 337 eligible infants (<28^0/7^ weeks gestation) who received a clinician-performed cardiac ultrasound between 6 and 12 h after birth. A web-central system classified PDAs as large or small based on the following cut-off: “large” = ductus diameter (mm) > 2.26 − (0.078 × postnatal age (h)).^20,21^ Infants with large PDAs were electronically randomized before they reached 12 h after birth to receive either ibuprofen or placebo. Infants with small PDAs were not randomized but enrolled in the small ductus group for follow-up. Parents, nurses, and physicians were unaware of treatment allocation as were the cardiologists or echocardiography-trained neonatologists reading the echocardiograms. Open-label ibuprofen back-up treatment was allowed in both groups if one or more pre-specified respiratory, cardiovascular, and/or echocardiographic “rescue” criteria were met (see TRIOCAPI study for details^20^). The median age of back-up treatment in both the placebo and early treatment group was 4 days. Rescue surgical ligation was used only if pharmacologic agents failed to constrict the PDA or were contraindicated.^20^ The decision to use rescue ligation was left to the attending neonatologist. Full details of the TRIOCAPI trial including screening, echocardiographic analyses, inclusion and exclusion criteria, enrollment, drug treatment protocols, rescue criteria, and definitions of study variables and outcomes have been published elsewhere.^20^

Echocardiographic studies were performed according to the trial’s study schedule for exams (days 3 and 14 after birth, before and after open-label treatment, and at 36 weeks corrected age). Additional echocardiograms were performed if there was a change in clinical symptoms indicative of a reopened PDA (systolic murmur or hyperdynamic precordium). The exam included two-dimensional imaging, M-mode, color flow mapping, and Doppler interrogation as previously described.^22^ Left atrium-to-aortic root ratio, ductus arteriosus diameter, mean, and end diastolic flow velocity of the left pulmonary artery were measured.^22^

A moderate-to-large PDA was defined by the presence of at least one of the following echocardiographic criteria: ductus diameter ≥1.5 mm (or PDA:left pulmonary artery diameter ratio ≥0.5), dilation of the left ventricular chamber (left atrium-to-aortic root ratio ≥1.5), pulsatile left–right ductal shunt (maximum velocity <2 m/s), left pulmonary artery diastolic flow velocity >0.2 m/s, diastolic flow absent or retrograde in the superior mesenteric artery, middle cerebral artery, or renal artery.^22–24^ PDAs that did not meet these criteria were considered to be “constricted” (small or closed).

Our primary outcome for the current study was the incidence of BPD-any grade as defined by the room air challenge test performed at 36 weeks postmenstrual age.^25^ We also examined the incidence of BPD or death prior to 36 weeks as a secondary outcome so that any infants who could not be evaluated for BPD because they died prior to the 36 weeks evaluation could be included.

### Statistical analyses

Our primary goal was to examine the effect of the duration of invasive tracheal ventilation on the relationship between the variable “duration of PDA exposure” and the outcome BPD. Prior observational studies have reported that infants <28 weeks gestation, who were exposed to a moderate-to-large PDA for longer than 7–14 days, had a significantly higher incidence of BPD than those exposed to shorter durations (the incidence of any-grade BPD (defined by the room air challenge test) appeared to be increased with PDA exposures ≥14 days,^16,18,19^ while the incidence of more severe grades of BPD, grades 2 and 3,^26^ increased with somewhat shorter PDA exposures (≥7 days).^16,18,19^ In addition, once the threshold exposure of 14 days was reached, additional exposures (>15 days) were not associated with additional increases in the incidence of BPD.^16,18,19^

In the previously reported secondary analysis of the PDA-TOLERATE trial that examined the interaction between duration of tracheal ventilation and duration of PDA exposure on the incidence of both BPD-any grade and BPD (grades 2 and 3), a duration of PDA exposure midway between ≥7 and ≥14 days (≥11 days) was used to define “prolonged PDA exposure.”^16,18,19^ However, in our current secondary analysis of the TRIOCAPI trial, we used a duration of PDA exposure ≥14 days to define “prolonged PDA exposure” since we only planned to examine the outcome BPD-any grade. (We were not able to examine the outcome BPD (grades 2 and 3) since the clinical practice among French neonatologists was to continue continuous positive airway pressure as the main respiratory support (rather than nasal cannula) late in the hospitalization, making it impossible to use the grading system defined by Jensen et al.^26^)

Therefore, in our current study, we defined the variable “duration of PDA exposure” as a binary categorical variable where infants who had a cumulative exposure to a moderate-to-large PDA of at least 14 days (i.e., those that were still present at 14 days and beyond) were compared with infants who were exposed to moderate-to-large PDAs that constricted before 14 days and remained constricted throughout the hospitalization, or who never developed a moderate-to-large shunt at all.

We defined the variable “duration of invasive tracheal ventilation” as a binary variable (cumulative tracheal intubation <10 and ≥10 days) since prior studies have shown that the variable “tracheal intubation ≥10 days” was both significantly associated with the outcome BPD^16,18^ and more strongly associated with the outcome BPD than other neonatal variables.^27^

Stata software (Release 16.1; StataCorp LP, College Station, Texas) was used for all statistical analyses. *χ*^2^ and Student’s *t* tests were used to compare groups for categorical and parametric variables, respectively. We used multivariable logistic regression to build statistical models that could adjust for the possible confounding effects of prenatal and postnatal demographic variables on the relationship between “duration of PDA exposure” and the outcome BPD. We first created a basic model for the outcome BPD that included our variable of interest “duration of PDA exposure” and the variable “gestational age ≤25 weeks.” Using these two variables, we performed a logistic regression to determine the odds ratio (OR) of BPD for the primary independent variable “duration of PDA exposure.”

Next, we added each of the demographic variables listed in Table 1 (except for the variable “duration of intubation ≥10 days”) to the basic model and reran the logistic regression to determine how much the odds ratio (OR) for the variable “duration of PDA exposure” was altered by the addition of the new variable to the basic model. If the addition of the new variable altered the OR for the association between “duration of PDA exposure” and BPD by >10% we considered it to be an “important demographic variable” that should be added to the *Final Adjusted model*. We repeated this step for each of the demographic variables in Table 1. We constructed the Final Adjusted model by adding all of the “important demographic variables” to the basic model. This final multivariable model was analyzed with logistic regression using generalized estimating equations techniques to account for clustering within the center (Table 2).

**Table 1:** Demographic characteristics of infants who were exposed to a moderate-to-large PDA shunt for <14 days or ≥14 days.

Variable	Duration of exposure to a moderate-to-large PDA		*P* value
	<14 days^a^	≥14 days^b^	
	*N* = 221	*N* = 86	
Prenatal variables			
Multiple gestations (%)	27	34	
Preeclampsia (%)	8	5	
Maternal diabetes (%)	2	0	
Chorioamnionitis (%)	10	12	
Betamethasone—any (%)	92	95	
Betamethasone ≥48 h (%)	73	70	
Cesarean section (%)	55	45	
Neonatal variables			
Gestation (weeks) (m ± s.d.)	26.0 ± 1.0	25.8 ± 1.0	0.052
Gestation ≤25 weeks (%)	28	45	0.006
Birthweight (g) (m ± s.d.)	874 ± 167	838 ± 163	0.088
Small for gestational age (%)	11	8	
Outborn (%)	6	13	0.074
Male (%)	50	48	
5-min Apgar ≤5 (%)	15	21	
Still intubated at 24 h (%)	67	73	
ICH (grades 3 or 4) (%)^c^	11	5	
Bacteremia (%)	56	66	
Early targeted ibuprofen (%)	36	24	0.066
Any pharmacologic PDA treatment (%)^d^	63	77	0.029
PDA ligation (%)	2	30	<0.001
NEC/SIP (%)	8	15	0.080
Duration of intubation ≥10 days (%)	33	62	<0.001
Outcomes			
BPD (%) (*n* = 266)^e^	26	56	<0.001
BPD or death before 36 weeks (%)	38	59	<0.001
Death before 36 weeks (%)	15	8	

*P* values, only *p* values ≤0.100 are reported.

*NEC/SIP* necrotizing enterocolitis/spontaneous intestinal perforation, *BPD* bronchopulmonary dysplasia.

^a^Ductus that had closed or become small before the 14 days echocardiogram.

^b^Ductus that were moderate to large during the first week and were still moderate to large on the 14 days echocardiogram.

^c^Serious intraventricular hemorrhages.

^d^Infants who received Early targeted ibuprofen and/or later pharmacologic PDA treatment.

^e^BPD was evaluated in 266 infants. Forty-one infants died before they could be evaluated at 36 weeks postmenstrual age: PDA exposure <14 days (*n* = 34) and PDA exposure ≥14 days (*n* = 7).

**Table 2:** Generalized estimating equation models examining the relationship between PDA exposure and BPD or BPD/death before 36 weeks.

Characteristic	BPD^a^ (*N* = 266)			BPD/death before 36 weeks^b^ (*N* = 307)		
	OR	95% CI	*P* value	OR	95% CI	*P* value
Final model without variable “duration of tracheal intubation”						
PDA duration						
<14 days	Ref.			Ref.		
≥14 days	2.65	(1.79, 3.91)	<0.001	2.14	(1.69, 2.71)	<0.001
Final Model including variable “duration of tracheal intubation”^c^						
PDA duration, intubation <10 days						
PDA duration						
<14 days	Ref.			Ref.		
≥14 days	1.49	(0.98, 2.26)	0.060	1.37	(0.86, 2.19)	0.180
PDA duration, intubation ≥10 days						
PDA duration						
<14 days	Ref.			Ref.		
≥14 days	3.00	(1.58–5.71)	0.001	2.41	(1.47–3.95)	<0.001

The Final models for BPD and BPD/death were adjusted for the demographic variables from Table 1 that were considered to be “important demographic variables” (see “Methods”).

^a^Adjusted for PDA duration, gestational age, infant still intubated at 24 h, and PDA ligation.

^b^Adjusted for PDA duration, gestational age, infant still intubated at 24 h, PDA ligation, and intracranial hemorrhage (grades 3 or 4).

^c^Models below include an interaction term between PDA duration and “duration of intubation”; ORs were obtained by varying the referent value of intubation (either <10 days or ≥10 days).

To determine if PDA exposure has different associations with BPD depending on the length of tracheal intubation, we added the variable “duration of tracheal intubation” plus an interaction term (between “duration of tracheal intubation” and “duration of PDA exposure”) to the *Final Adjusted model* and reran the logistic regression using generalized estimating equations techniques (Table 2).

In addition to the “interaction” models just described, we also examined the effects of duration of PDA exposure on our outcome of interest in stratified models, where the total population was stratified into two subgroups based on their duration of intubation (either <10 days or ≥10 days) (Table 3). Logistic regression analyses, using generalized estimating equations techniques, were performed on each individual subgroup.

**Table 3:** Stratified generalized estimating equation models examining the relationship between PDA exposure and BPD or BPD/death before 36 weeks in two subpopulations of infants: those intubated for <10 days and those intubated for ≥10 days.

PDA duration among infants intubated <10 days						
Characteristic	BPD^a^ (*N* = 161)			BPD/death before 36 weeks^b^ (*N* = 181)		
	OR	95% CI	*P* value	OR	95% CI	*P* value
PDA duration						
<14 days	Ref.			Ref.		
≥14 days	1.34	(0.87, 2.08)	0.180	1.32	(0.75, 2.29)	0.324

PDA duration among infants intubated ≥10 days						
	BPD^a^ (*N* = 105)			BPD/death before 36 weeks^b^ (*N* = 126)		
PDA duration						
<14 days	Ref.			Ref.		
≥14 days	2.80	(1.31, 5.97)	0.008	2.17	(1.17, 4.02)	0.014

The Final models for BPD and BPD/death were adjusted for the demographic variables from Table 1 that were considered to be “important demographic variables” (see “Methods”).

^a^Adjusted for PDA duration, gestational age, infant still intubated at 24 h, and PDA ligation.

^b^Adjusted for PDA duration, gestational age, infant still intubated at 24 h, PDA ligation, and intracranial hemorrhage (grades 3 or 4).

In order to examine the effects of the variable “duration of tracheal intubation” on the relationship between PDA exposure and the outcome “BPD or Death prior to 36 weeks”, we used the same stepwise approach described above for BPD,

## Results

**Fig. 1:**
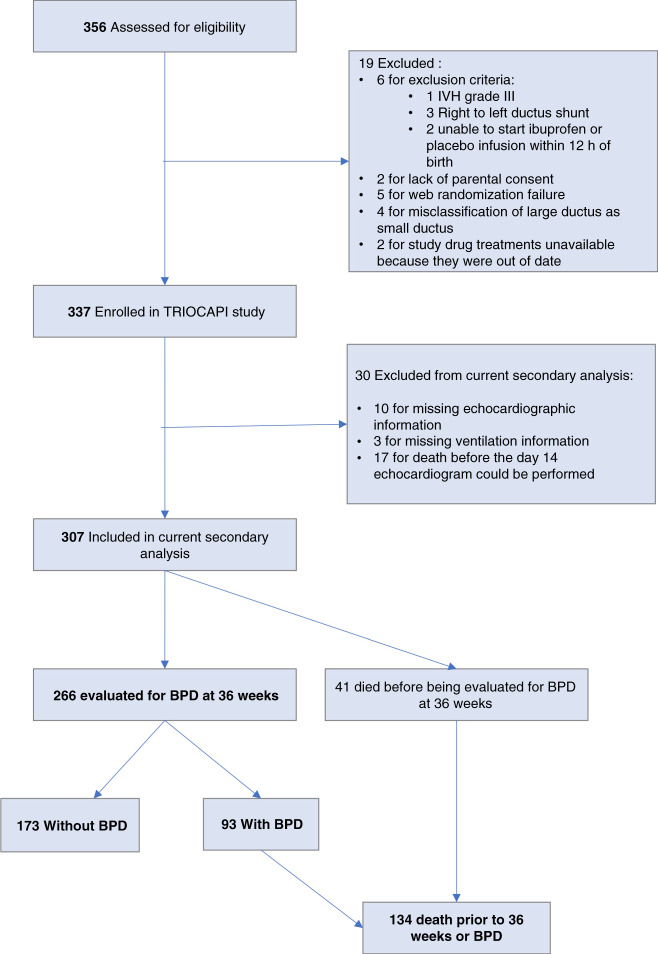
Flow diagram of patient distribution in the current TRIOCAPI trial secondary analysis.

Among the 337 infants enrolled in the placebo (*n* = 114), early ibuprofen (*n* = 114), and initial small ductus (*n* = 109) arms of the TRIOCAPI trial, complete information was available from 307 infants for our current study (30 infants had incomplete study information: 10 infants had missing echocardiographic information, 3 infants had missing ventilation information, and 17 infants died before the day 14 echocardiogram could be performed) (Fig. 1).

Among the remaining 307 study infants, 221 were exposed to a moderate-to-large PDA for <14 days and 86 for ≥14 days. Forty-one infants died before being evaluated for BPD at 36 weeks (Table 1). There was no significant difference in the death rates prior to 36 weeks between infants exposed to a moderate-to-large PDA for <14 days and those exposed for ≥14 days (Table 1).

Among the infants evaluated for BPD at 36 weeks, 35% (93/266) had BPD. Infants who were exposed to a moderate-to-large PDA for ≥14 days had a significantly higher incidence of BPD than those who were exposed to shorter periods (Table 1). We created multivariable models to adjust for the possible confounding effects of the demographic characteristics listed in Table 1 on the relationship between PDA exposure and the incidence of BPD (see “Methods”) (Table 2). A significant relationship between prolonged PDA exposure (≥14 days) and the incidence of BPD persisted even after adjusting for all of the “important demographic covariates” in the Final adjusted model (Table 2—Final Model without variable “duration of tracheal intubation”).

Our main objective was to determine if the amount of invasive respiratory support that infants required (tracheal intubation <10 or ≥10 days) affected the relationship between PDA exposure and BPD. When we added the variable “duration of intubation” plus a term for the interaction between “duration of PDA exposure” and “duration of intubation” to the statistical models, we found that the association between PDA exposure and BPD was different depending on the duration of intubation (Table 2). Infants who required tracheal intubation for <10 days had similar rates of BPD, whether the ductus constricted during the first week or whether it persisted beyond 14 days (OR for PDA duration (95% confidence interval (CI)) = 1.49 (0.98, 2.26)). In contrast, when infants required tracheal intubation for ≥10 days, exposure to a persistent PDA for ≥14 days was associated with a significant increase in the risk of developing BPD (OR for PDA duration = 3.00 (1.58, 5.71)) (Table 2). These findings were even more apparent when the total population was divided and stratified into two separate subgroups based on their duration of intubation (either <10 days (OR for PDA duration = 1.34 (0.87, 2.08)) or ≥10 days (OR for PDA duration = 2.80 (1.31, 5.97)) and the multivariable regression analyses for BPD were performed on each individual subgroup (Table 3).

We also examined the effects of invasive respiratory support on the relationship between PDA exposure and the outcome BPD or death prior to 36 weeks, to include any infants who could not be evaluated for BPD because they died prior to the 36 weeks evaluation. As we saw for BPD alone, persistent, moderate-to-large PDAs (for ≥14 days) were not associated with an increased risk of BPD or death when infants required <10 days of intubation. However, if infants required intubation for ≥10 days, prolonged PDA exposures were associated with an increased risk of BPD or Death (see Tables 2 and 3).

## Discussion

Our secondary analysis of the multicenter TRIOCAPI trial agrees with the results of the prior single-center observational studies that found an association between the duration of PDA exposure and the incidence of BPD;^14–18^ they extend the prior studies’ findings to more narrowly identify which infants with a moderate-to-large PDA are at greatest risk for developing BPD. In our study prolonged PDA exposure (≥14 days) was associated with an increased incidence of BPD only in infants who also received prolonged tracheal ventilation (≥10 days). The incidence of BPD among infants who received less ventilatory support (intubation for <10 days) was the same whether the ductus closed shortly after birth or whether it persisted as a moderate-to-large shunt beyond 14 days. While our results do not prove a cause-and-effect relationship, they do indicate that the presence of a moderate-to-large PDA shunt that persists beyond 14 days in infants requiring prolonged intubation may be a useful clinical marker for identifying infants at increased risk for BPD. Conversely, our results suggest that if the only goal of prophylactic or early routine ductus closure is to decrease the incidence of BPD, then early PDA closure may be unnecessary in infants who require shorter durations of intubation (<10 days) since it does not seem to alter the incidence of BPD.

Our study has several limitations. As an observational study, it cannot distinguish between causation and association. Even though we adjusted our analyses for important demographic variables, unmeasured differences in practice might have affected the rates of BPD and BPD or Death before 36 weeks. In addition, the relatively small size of our study may have made it difficult to detect significant differences among some of our PDA exposure subgroups. We focused our study on infants who survived to 14 days and continued to have a persistent PDA beyond 14 days and did not address whether shorter exposures to a moderate-to-large PDA during the first 2 weeks or longer exposures, beyond 14 days, could have altered our results. Several prior RCTs have examined the effects of shorter exposures to a PDA, during the first 2 weeks, and found no noticeable effect on the incidence of BPD.^5,8,9,11,28^ Similarly, three recent observational studies have reported that although the incidence of BPD did not appear to be increased until infants were exposed to a moderate-to-large PDA for at least 14 days, once this threshold was reached, additional exposures (>15 days) were not associated with additional increases in the incidence of BPD.^16,18,19^

Our study contains useful information for designing a future RCT with the goal of determining whether early PDA closure can alter the incidence of BPD. We suggest that such a trial would require more restrictive enrollment criteria than previous trials, focusing on infants who are intubated at the time of enrollment and likely to remain intubated for ≥10 days (in addition to having a moderate-to-large PDA shunt that is likely to persist for at least 14 days if left untreated). Our findings suggest that recruiting infants without taking into account their need for invasive respiratory support may conceal potentially important effects of early PDA closure by diluting the at-risk population (those requiring intubation for ≥10 days) with infants requiring shorter durations of tracheal intubation, for whom closing the PDA appears to have little or no consequence. In addition, for the study to achieve its goal, with the fewest number of enrollees, one group would need to receive an effective treatment that could guarantee ductus constriction within 7–10 days of birth in ≥80% of the treated infants, while the other group would need to remain untreated, allowing the moderate-to-large shunt to persist for at least 14 days.

In conclusion, in the TRIOCAPI trial, the presence of a moderate-to-large PDA shunt was associated with an increased risk of BPD when it persisted beyond 14 days and the infant also required prolonged intubation (≥10 days). On the other hand, prolonged exposure to a PDA did not appear to be associated with an increased risk of BPD if the infant only required <10 days of intubation.

## Supplementary information

Appendix

Supplementary information

## Data Availability

The datasets generated and/or analyzed during the current study are available from the corresponding author on reasonable request.
