# Supplementary material for: Patent ductus arteriosus, tracheal ventilation, and the risk of bronchopulmonary dysplasia
Source: Pediatr Res. 2021 Mar 31;91(3):652–8. doi: 10.1038/s41390-021-01475-w (PMC8904244; doi:10.1038/s41390-021-01475-w)
Supplement: Supplementary file 1 — Appendix [file 41390_2021_1475_MOESM1_ESM.pdf]

THE JOURNAL OF PEDIATRICS • www.jpeds.com

Effect of Early Targeted Treatment of Ductus Arteriosus with Ibuprofen on Survival Without Cerebral Palsy at 2 Years in Infants with Extreme Prematurity: A Randomized Clinical Trial

Jean-Christophe Rozé, MD, PhD[1,3], Gilles Cambonie, MD, PhD[4], Aurelie Le Thuaut, MS[2], Thierry Debillon, MD, PhD[5], Isabelle Ligi, MD, PhD[6], Geraldine Gascoin, MD, PhD[7], Juliana Patkai, MD[8], Alain Beuchee, MD, PhD[9], Geraldine Favrais, MD, PhD[10], Cyril Flamant, MD, PhD[1,3], Xavier Durrmeyer, MD, PhD[11,12], and Ronald Clyman, MD[13]

Objective To examine the effects of early echocardiography-targeted ibuprofen treatment of large patent ductus arteriosus (PDA) on survival without cerebral palsy at 24 months of corrected age.

Study design We enrolled infants born at <28 weeks of gestation with a large PDA on echocardiography at 6-12 hours after birth to ibuprofen or placebo by 12 hours of age in a multicenter, double blind, randomized-controlled trial. Open-label ibuprofen was allowed for prespecified criteria of a hemodynamically significant PDA. The primary outcome was survival without cerebral palsy at 24 months of corrected age.

Results Among 337 enrolled infants, 109 had a small or closed ductus and constituted a reference group; 228 had a large PDA and were randomized. The primary outcome was assessed at 2 years in 108 of 114 (94.7%) and 102 of 114 (89.5%) patients allocated to ibuprofen or placebo, respectively. Survival without cerebral palsy occurred in 77 of 108 (71.3%) after ibuprofen, 73 of 102 (71.6%) after placebo (adjusted relative risk 0.98, 95% CI 0.83-1.16, *P* = .83), and 77 of 101 (76.2%) in reference group. Infants treated with ibuprofen had a lower incidence of PDA at day 3. Severe pulmonary hemorrhage during the first 3 days occurred in 2 of 114 (1.8%) infants treated with ibuprofen and 9 of 114 (7.9%) infants treated with placebo (adjusted relative risk 0.22, 95% CI 0.05-1.00, *P* = .05). Open-label rescue treatment with ibuprofen occurred in 62.3% of infants treated with placebo and 17.5% of infants treated with ibuprofen (*P* < .001), at a median (IQR) age of 4 (3, 5) and 4 (4, 12) days, respectively.

Conclusions Early echocardiography-targeted ibuprofen treatment of a large PDA did not change the rate of survival without cerebral palsy. *(J Pediatr 2020;■:1-10)*.

Trial registration Eudract 2011-003063-30 and ClinicalTrials.gov: NCT01630278.

See editorial, p •••

Uncertainty and controversy still exist about the significance, evaluation, and management of patent ductus arteriosus (PDA) in infants born preterm.[1-5] Prophylactic treatment with indomethacin or ibuprofen reduces the risk of developing a subsequent PDA and the need for surgical ligation. However, it does not reduce in-hospital mortality or morbidity[6-8] and unnecessarily exposes a large proportion of infants to drugs that have side effects (such as pulmonary hypertension)[9] without conferring significant long-term benefits. Current evidence does not support the use of prophylactic indomethacin[10] or ibuprofen[11] for the prevention of morbidities associated with the presence of a PDA. However, most of the trials that explored the effects of early ductus closure on later morbidities enrolled patients based on whether the PDA was "present or absent," without taking into account the magnitude of the left-to-right shunt.[12,13] Recent studies have

ASQ	Ages and Stages Questionnaires
aRR	Adjusted relative ratio
IVH	Intraventricular hemorrhage
NICU	Neonatal intensive care unit
PDA	Patent ductus arteriosus
RCT	Randomized clinical trial

From the Departments of [1]Neonatology, and [2]biostatistics, Nantes University Hospital, Nantes, France; [3]Centre d'Investigation Clinique CIC1413, INSERM-Nantes University Hospital, Nantes, France; [4]Neonatal Medicine, Montpellier University Hospital, Montpellier, France; [5]Department of Neonatology, University Hospital of Grenoble, Grenoble, France; [6]Department of Neonatology, Assistance Publique Hôpitaux de Marseille, Marseille, France; [7]Neonatal Medicine, Angers University Hospital, Angers, France; [8]Neonatal Intensive Care Unit, Cochin Hospital, Maternity of Port-Royal, Paris, France; [9]Department of Neonatology, Rennes University Hospital, Rennes, France. [10]Department of Neonatology, Tours University Hospital, Tours, France; [11]Department of Neonatology, Centre Hospitalier Intercommunal de Créteil, Créteil, France; [12]Université Paris Est Créteil, Faculté de Médecine de Créteil, IMRB, GRC CARMAS, Créteil, France; and [13]Departments of Pediatrics, and Cardiovascular Research Institute, University of California San Francisco, San Francisco, CA.

Funded by Programme Hospitalier de Recherche Clinique 2010 of the French Ministry of Social Affairs and Health. The funders of the study had no role in study design, data collection or analysis, preparation of the manuscript, or decision to publish. The authors declare no conflicts of interest.

0022-3476/$ - see front matter. © 2020 Elsevier Inc. All rights reserved.
https://doi.org/10.1016/j.jpeds.2020.12.008

shown that neonatal morbidities associated with persistent PDA are only associated with hemodynamically significant moderate-to-large PDAs, not with small, nonsignificant PDAs.[14-17]

As a result, the identification of infants with large ductal diameters (large PDA) soon after birth has been proposed as a biomarker that might be used to identify and initiate targeted treatment of infants at greatest risk for developing a significant PDA, reducing the number of infants who receive treatment unnecessarily. This approach was tested in the Ductal Echocardiographic Targeting and Early Closure Trial randomized clinical trial (RCT),[18] performed in Australia, which showed evidence for protection from pulmonary hemorrhage, reduction in the need for subsequent PDA treatment and a trend toward reduced severe brain lesions among infants treated with indomethacin. Unfortunately, this trial was prematurely halted due to disruption in the supply of indomethacin for the trial.

Therefore, we designed the following multicenter RCT using early cardiac ultrasound to guide management of a PDA. The primary goal of our RCT was to assess the effects of early cardiac ultrasound-targeted ibuprofen treatment of a large PDA on survival without cerebral palsy at 24 months of corrected age. In France, ibuprofen, rather than indomethacin, is approved for PDA treatment. Even though prophylactic ibuprofen has not been shown to reduce the incidence of intraventricular hemorrhage (IVH) as has been shown for indomethacin,[6-8] we hypothesized that the early elimination of a PDA shunt with ibuprofen might improve long-term neurodevelopmental outcomes.

Methods

The Targerted by Echocardiographic Treatment of the Ductus Arteriosus in Preterm Infants by Ibuprofen study was a double-blind, multicenter, randomized, placebo-controlled, clinical trial performed at 11 French tertiary-care neonatal intensive care units (NICUs) from 2012 to 2017. The trial was approved by the national ethics committee (Comite de Protection des Personnes Ouest IV) and other French national agencies and registered with Eurodract database (2011-003063-30) as well as ClinicalTrials.gov (NCT00623740). Parents gave written informed consent. The trial was monitored by an independent data and safety monitoring board.

Infants were eligible if they delivered between $24^{0/7}$ and $27^{6/7}$ weeks of gestation. Infants were excluded if they were likely to die soon after birth or if they had any of the following: IVH grade III or IV during the enrollment evaluation,[19] a major congenital anomaly, right to left ductus arteriosus shunt for more than one-third of the cardiac cycle suggesting pulmonary hypertension, platelet count <50 000/mL, maternal use of ibuprofen within the last 6 weeks, unable to start study treatments within 12 hours of birth, and unlikely to appear for 24-month visit.

Randomization, Concealment, and Masking

A cardiac ultrasound was performed on eligible infants by certified neonatologists[20] at 6-12 hours after birth to establish structural normality, measure the PDA diameter,[21] and assess the direction of ductal shunt. A video was produced and distributed to each investigator by Professor Gournay to standardize the cardiac ultrasound examination. A web-central system classified PDAs as large or small based on the following cut-off: "large" = ductus diameter in mm $>2.26 - (0.078 \times \text{postnatal age in hours})$.[22] Only infants with a large PDA were randomized. Infants with a small PDA were not randomized but enrolled in the small ductus reference group for follow-up.

Randomized infants were assigned 1:1 to either ibuprofen or placebo via a central computer-generated list with a fixed block size of 4, stratified by gestational age groups ($24^{0/7}$- $25^{6/7}$ and $26^{0/7}$-$27^{6/7}$ weeks) and center. In case of twins, each infant was randomized separately. The appearance of the ibuprofen and placebo vials was identical. Study investigators, parents, physicians during the hospitalization, and those performing follow-up examinations, nurses, and external statisticians were unaware of treatment allocation.

Intervention

Investigational drugs were supplied as blinded treatment by LC2 clinical batch packaging and logistics laboratory, (LC2 Pharmaceuticals). Vials contained either 5 mg/mL intravenous ibuprofen, Pedea, Orphan Europe (a racemic mixture of R-iburprofen and S-ibuprofen, detailed information about this formulation and pharmacokinetics is available on the European medicines Agency website: http://emea.europa.eu) or 0.9% saline as placebo. Infants received an intravenous loading dose of 2 mL/kg of either placebo or ibuprofen (10 mg/kg) between 6 and 12 hours after birth, followed by 2 injections of 1 mL/kg, 24 and 48 hours after the first injection of placebo or ibuprofen (5 mg/kg). In addition to echocardiograms performed for routine clinical care of the infants, 3 study echocardiograms were performed at day 3, day 14, and at 36 weeks of postmenstrual age. Study echocardiograms included the following measurements: left atrial to aortic root ratio, ductus arteriosus diameter, mean and end diastolic flow velocity of the left pulmonary artery, ductus arteriosus, descending aorta, and either the superior mesenteric artery, middle cerebral artery, or renal artery.[23]

Open-label ibuprofen back-up treatment was allowed in both groups before or after the third postnatal day based on the following criteria: before day 3, severe pulmonary hemorrhage or severe hypotension (definitions, **Table I**; available at www.jpeds.com) plus echocardiographic evidence of a hemodynamically significant ductus arteriosus left to right shunt (described below). After day 3, open-label treatment was limited to infants with a hemodynamically significant ductus arteriosus defined by the need for positive pressure ventilation, nasal continuous positive airway pressure, or persistent severe hypotension plus the presence of at least 1 of the following echocardiographic criteria: ductus diameter ≥1.5 mm,

expansion of the left ventricular chamber (left atrial to aortic root ratio ≥1.5), pulsatile left-right ductal shunt (maximum velocity <2 m/second), diastolic flow absent or retrograde in the superior mesenteric artery, middle cerebral artery, or renal artery.[24] Open-label ibuprofen daily doses were 10, 5, and 5 mg/kg; 14, 7, and 7 mg/kg, and 19, 9, and 9 mg/kg over 3 days, for infants <70, 70-108, and >108 hours of postnatal age, respectively.[25]

Primary Outcome

The primary outcome was survival without cerebral palsy at 24 months of corrected age. Infants were examined by local pediatricians trained in the Amiel-Tison examination. Cerebral palsy was defined using the Amiel-Tison classification,[26,27] which includes all forms of cerebral palsy from minimal cerebral palsy (with uni- or bilateral tonic stretch reflexes with the ability to walk independently at 2 years of age)[28] to a severe form (without independent walking). The severity of cerebral palsy was graded according to the Gross Motor Function Classification System.[29] The forms from the follow-up consultation were sent to the national coordination center where 2 physicians reviewed the concordance between the form items and the diagnosis of "cerebral palsy (yes/no)" proposed by the local examiner and made a final decision. In case of disagreement, a third referent decided the outcome. All referents were unaware of the participants' study drug allocation.

Secondary Outcomes

Secondary outcomes (Table I) included ductus status on days 3 and 14, open-label rescue treatment, surgical ligation, severe morbidities,[19,30-33] death and survival without severe morbidity at 36 weeks of postmenstrual age or discharge, whichever came first. We also compared the incidences of and duration of mechanical ventilation, noninvasive ventilation, and oxygen delivery between the treatment approaches. At 24 months of corrected age, the second version of the French translation of the Ages and Stages Questionnaire (ASQ)[34] was completed by parents. We evaluated the total ASQ score, the score below threshold (<186, based on our previous publications[35,36]), the ASQ domain subscores (communication, gross motor, fine motor, problem solving, and personal-social domains), and their subscores below threshold (<36.5, 36.0, 36.4, 32.9, and 35.6, respectively).

Statistical Analyses

Based on data from the EPIPAGE 1 cohort,[37] we hypothesized that early treatment with ibuprofen should result in an increase in survival without cerebral palsy of 18% (from 35% to 53%) in infants examined at 2 years, Thus, assuming a 10% loss of follow-up, 115 patients should be randomized per arm (α-error of 0.05 and [power 1-β error] at 80%). Because only 60% of the patients were estimated to have a large PDA, we planned to include a total of 385 (230 ÷ 0.6) patients. However, the sponsor stopped the trial after enrolling 337 infants (356 assessed for eligibility, Figure) as the proportion of patients with a large PDA was 68%.

Our primary analysis used a modified intention-to-treat approach including all infants who were randomized and received the first study dose. The primary outcome was survival without cerebral palsy at 24 months of corrected age. Infants in the small ductus group were analyzed as reference. Primary outcomes were assessed using generalized estimating equations. Treatment effects were summarized with the use of adjusted relative risks and 95% CIs estimated with a log-link function and compound symmetric correlation structure accounting for multiple births. All analyses were adjusted for gestational age at birth as fixed effect, recruitment site as a random effect, and accounting for clustering of siblings from the same pregnancy.

As sensitivity analyses, we performed multiple imputations with the use of chained equations separately in each group to address missing data under a missing-at-random assumption. We also performed a per-protocol analysis with and without imputations. All sensitivity analyses were performed with log-binomial, log-normal, or log-Poisson generalized estimating equation models as appropriate. Results were considered exploratory and were not adjusted for multiple testing.

In addition to sensitivity analyses, planned subgroup analyses were performed among infants with birth weight z scores < -1 for the primary outcome and to compare infants from the placebo group with those from the small ductus group. In a second complementary analysis, we compared our reference group (infants with a small ductus) with the placebo-treated large ductus group to calculate the sensitivity, specificity, and positive and negative likelihood ratio of the biomarker "ductus diameter measurements before 12 hours" to discriminate between infants who would and would not subsequently meet the study's criteria for a hemodynamically significant PDA. Statistical analyses were performed with SAS v 9.4 (SAS Institute, Inc).

Results

We recruited 349 infants from March 26, 2012 to February 2, 2017, from 11 French tertiary-care NICUs. Among them, 337 were enrolled in the modified intention-to-treat analysis (Figure). Ductus arteriosus assessed by echocardiography (at less than 12 hours postnatal age) was assessed as small or closed in 109 infants and large in 228 infants. Gestational age, ductus diameter, and end-diastolic velocity in left pulmonary artery were significantly different between small and large ductus diameter groups, respectively: mean (SD) 26.1 (1.0) vs 25.9 (1.0) weeks, $P = .03$; 1.16 (0.48) vs 2.27 (0.52) mm, $P < .001$; and 0.13 (0.16) vs 0.19 (0.12) m/s, $P = .001$.

The 228 infants classified with a large ductus were randomized to ibuprofen (n = 114) or placebo (n = 114). Baseline characteristics of mothers and infants were well balanced between the 2 groups (Table II).

ARTICLE IN PRESS

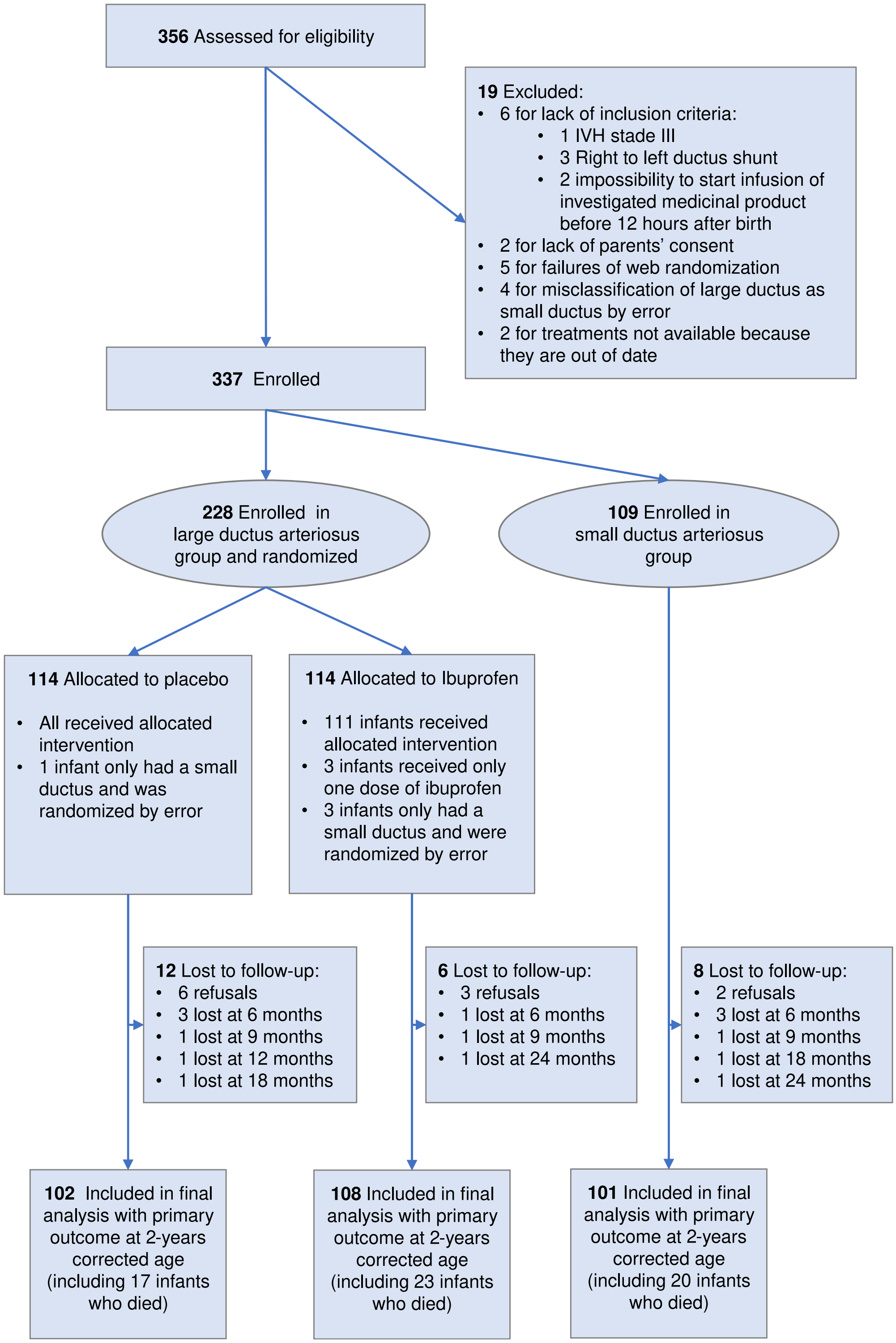

Figure. Consort[38] diagram of study population.

Primary and Secondary Outcomes

The primary outcome was assessed in 108 of 114 (94.7%) and 102 of 114 (89.5%) patients allocated to ibuprofen or placebo, respectively (**Figure**). In the ibuprofen group 77 of 108 (71.3%) infants were alive without cerebral palsy at 24 months of corrected age compared with 73 of 102 (71.6%) in the placebo groups: adjusted relative risk (aRR) 0.98; 95% CI 0.83-1.16, P = .83; in comparison 77 of 101 (76.2%) of the infants in the small ductus arteriosus group were alive without cerebral palsy at 24 months (**Table III**).

Table II. Baseline characteristics

Characteristics	Large ductus group Placebo n = 114	Large ductus group Ibuprofen n = 114	Small ductus group n = 109	*P* value small vs large (placebo + ibuprofen)
Maternal characteristics				
No. with data (% of recruited patients)	99 (100)	99 (100)	94 (100)	
Mean age, y (SD)	29.7 (6.2)	30.9 (5.6)	30.2 (5.1)	.95
Education				
High school or less, n (%)	22 (22.2)	17 (17.2)	14 (14.9)	.02
Some college, n (%)	11 (11.1)	12 (12.1)	3 (3.2)	
College degree or greater, n (%)	17 (17.2)	20 (20.2)	14 (14.9)	
Not known or not reported, n (%)	49 (49.5)	50 (50.5)	63 (67.0)	
Primiparity, n (%)	47 (47.5)	53 (53.5)	45 (45.0)	.67
Infant characteristics				
No. with data (% of recruited patients)	114 (100)	114 (100)	109 (100)	
Multiple gestation, n (%)	32 (28.1)	31 (27.2)	35 (32.1)	.40
Received prenatal glucocorticoids, n (%)	108 (94.7)	106 (93.0)	102 (93.6)	.91
Birth after tocolysis, n (%)	70 (61.4)	67 (58.8)	72 (66.1)	.29
Delivery in the same hospital as the NICU, n (%)	105 (92.1)	104 (91.2)	104 (95.4)	.21
Cesarean delivery, n (%)	65 (57.0)	60 (52.6)	54 (49.5)	.36
Cesarean delivery before labor, n (%)	39 (34.2)	36 (31.6)	26 (23.9)	.09
Abnormal fetal heart rate monitoring, n (%)	46 (40.4)	37 (32.5)	28 (25.7)	.05
Mean gestational age, wk (SD)	25.9 (1.00)	25.8 (1.04)	26.1 (0.97)	.03
24 wk, n (%)	12 (10.5)	15 (13.2)	8 (7.3)	
25 wk, n (%)	29 (25.4)	27 (23.7)	22 (20.2)	
26 wk, n (%)	35 (30.7)	34 (29.8)	29 (26.6)	
27 wk, n (%)	38 (33.3)	38 (33.3)	50 (45.9)	
Mean birth weight, g (SD)	870 (175)	850 (165)	860 (165)	.99
Birth weight z score (SD)	0.15 (0.96)	0.03 (0.90)	−0.07 (0.95)	.13
Male sex, n (%)	60 (52.6)	53 (46.5)	55 (50.5)	.88
1-min Apgar score, median (IQR)	5 (2, 8)	6 (3, 8)	5 (2, 8)	.88
5-min Apgar score, median (IQR)	8 (6, 10)	8 (7, 10)	8 (6, 10)	.82
5-min Apgar score <5, n (%)	12 (10.7)	10 (8.9)	12 (11.0)	.74
First cerebral ultrasound before randomization:				
Cerebral ultrasound results not available, n (%)	1 (0.9)	1 (0.9)	1 (0.9)	.70
Normal, n (%)	101 (88.6)	97 (85.1)	98 (89.9)	
IVH grade I or II, n (%)	12 (10.5)	16 (14.0)	10 (9.2)	
First echocardiography before randomization:				
No. with data (% of recruited patients)	114 (100)	114 (100)	109 (100)	
Mean postnatal age at first echocardiography, h (SD)	9.0 (2.0)	8.8 (2.0)	8.4 (2.0)	.04
Mean ductus arteriosus' diameter, mm (SD)	2.28 (0.54)	2.25 (0.50)	1.16 (0.48)	<.001
Mean left atrial/aortic root ratio (SD)	1.36 (0.28)	1.35 (0.30)	1.28 (0.32)	.01
Mean of mean left pulmonary artery velocity, m/s (SD)	0.34 (0.14)	0.33 (0.10)	0.32 (0.17)	.02
Mean telediastolic left pulmonary artery velocity, m/s (SD)	0.18 (0.12)	0.20 (0.13)	0.13 (0.16)	<.001

For secondary outcomes, the ductus arteriosus was more often observed as closed at day 3 in the ibuprofen group (**Table IV**). Mean and end-telediastolic left pulmonary artery flow were also significantly lower on day 3 in the ibuprofen group. Open-label rescue treatment with ibuprofen occurred more frequently in the placebo group (P < .001) (**Table IV**). The median (IQR) age of first rescue treatment was 4 (3,5) days in the placebo group and 4 (4, 12) days in the ibuprofen group (**Table IV**). There was no difference in the rate of surgical ligation between the 2 groups (**Table IV**).

Survival analysis with the use of the unadjusted Kaplan-Meier method and a Cox proportional-hazards model adjusted for gestational age and center produced similar results (hazard ratio, 1.52; 95% CI 0.80-2.89; P = .20). Survival without morbidity at 36 weeks of postmenstrual age was not significantly different between the placebo and ibuprofen groups (**Table III**).

The ASQ was assessed at 2 years of corrected age in 78 infants in each group. Thirty-nine infants (50%) in each group had no domain at risk. Only one domain's score, problem solving abilities, was significantly lower in placebo-treated infants compared with ibuprofen-treated infants (P = .003) (**Table III**). There was also a trend for placebo-treated infants to have a lower fine motor skills scores and a higher rate of total ASQ scores <186 (**Table III**).

Adverse Events

Adverse events were reported for 102 of 114 (89.5%) and 93 of 114 (81.6%) infants in the ibuprofen and placebo groups, respectively (P = .68) (**Table V**; available at www.jpeds.com). Pulmonary hemorrhage during the first 3 postnatal days was observed in 2 of 114 (1.8%) and 9 of 114 (7.9%) infants in the ibuprofen and placebo groups, respectively (aRR 0.22, 95% CI 0.05-1.00), P = .05). Isolated gastrointestinal perforation occurred in 10 of 114 (8.8%) and 4 of 114 (3.5%) infants

Table III. Primary and secondary exploratory outcomes

	Large ductus group				Small ductus group		
Outcomes	Placebo n = 114	Ibuprofen n = 114	aRRs [95% CI]*	*P* value Ibuprofen vs placebo	n = 109	*P* value vs placebo†	*P*-value vs Ibuprofen‡
Primary outcome at 2 y of corrected age							
No. with data available, n (%)	102 (89.5)	108 (94.7)			101 (92.7)		
Survival without cerebral palsy, n (%)	73 (71.6)	77 (71.3)	0.98 [0.83-1.16]	.83	77 (76.2)	.63	.49
Death, n (%)	17 (16.7)	23 (21.3)	1.25 [0.72-2.19]	.42	20 (19.8)	.33	.82
Cerebral palsy, n (%)	12 (11.8)	8 (7.4)	0.62 [0.26-1.45]	.27	4 (3.7)	.05	.30
Gross motor function classification system, stage 1 to 2, n (%)	9 (8.8)	6 (5.3)	-	-	2 (1.8)	-	-
Gross motor function classification system, stage 3 to 4, n (%)	3 (2.9)	2 (1.8)	-	-	2 (1.8)	-	-
Secondary exploratory outcomes							
Outcomes at 36 wk of corrected age							
No. with data available (%)	114 (100)	114 (100)	-	-	109 (100)	-	-
Death, n (%)	16 (14.0)	23 (20.2)	1.43 [0.80-2.53]	.22	19 (17.4)	.30	.89
Survival without morbidity,§ n (%)	50 (43.9)	48 (42.1)	0.94 [0.71-1.26]	.70	50 (45.9)	.96	.66
Respiratory course							
No. with data available (%)	114 (100)	114 (100)	-	-	109 (100)	-	-
Median (IQR) cumulative duration of mechanical ventilation, d	7.5 (1, 22)	5 (1, 17)	-	.26	3 (1, 15)	.02	.12
Median (IQR) cumulative duration of noninvasive ventilation, d	40 (25, 40)	39 (21, 59)	-	.47	43 (26.5, 56.5)	.87	.37
Median (IQR) cumulative duration of oxygen delivery, d	39 (11, 66)	29 (8, 52)	-	.16	21 (10, 46)	.03	.41
ASQ results at 24 mo of corrected age							
No. of children with data available (%)	78 (68.4)	78 (68.4)	-	-	72 (66)	-	-
All ASQ domains above threshold,¶ n (%)	39 (50.0)	39 (50.0)	0.98 [0.72-1.34]**	.91**	42 (58.3)	.41**	.34**
One ASQ domain below threshold,¶ n (%)	16 (20.5)	24 (30.8)			14 (19.4)		
Two or more ASQ domains below threshold,¶ n (%)	23 (29.5)	15 (19.2)			16 (22.2)		
Mean total ASQ score (SD)	224.9 (55.5)	236.3 (46.2)	-	.15	240.4 (39.7)	.06	.65
No. of children with total ASQ score <186, n (%)	15 (19.2)	7 (9.0)	0.44 [0.19-1.03]	.06	5 (6.9)	.04	.78
Mean fine motor skills score (SD)	46.9 (11.4)	49.7 (8.6)	-	.07	50.4 (9.3)	.06	.86
Fine motor skills below threshold,¶ n (%)	13 (16.7)	8 (10.3)	0.59 [0.26-1.33]	.20	8 (11.1)	.37	.75
Mean problem solving abilities score (SD)	42.8 (13.4)	48.8 (11.2)	-	.003	46.5 (11.6)	.07	.22
Problem solving abilities domain below threshold,‡ n (%)	17 (21.8)	8 (10.3)	0.47 [0.21-1.03]	.06	9 (12.5)	.14	.68
Mean personal social skills score	44.5 (12.8)	45.2 (10.6)	-	.71	46.4 (10.0)	.36	.56
Mean personal social skills domain below threshold,‡ n (%)	19 (24.4)	16 (20.5)	0.83 [0.46-1.48]	.52	13 (18.1)	.41	.85

*Relative risks are expressed for ibuprofen vs placebo and were generated with the use of generalized estimating equation models adjusted for gestational age at birth and recruitment site and accounting for clustering of siblings from the same pregnancy.
†*P* values are expressed for small ductus vs placebo and were generated with the use of generalized estimating equation models adjusted for gestational age at birth and recruitment site and accounting for clustering of siblings from the same pregnancy.
‡*P* values are expressed for small ductus vs ibuprofen and were generated with the use of generalized estimating equation models adjusted for gestational age at birth and recruitment site and accounting for clustering of siblings from the same pregnancy.
§Morbidities at 36 weeks of corrected age included bronchopulmonary dysplasia, necrotizing enterocolitis, grade III-IV IVH, or periventricular leukomalacia.
¶Thresholds for communication, gross motor, fine motor, problem solving and personal-social domains are 36.5, 36.0, 36.4, 32.9, and 35.6, respectively.
**aRR and *P* values for no domain vs 1 or more domain below threshold.

in the ibuprofen and placebo groups, respectively (aRR 2.46 [0.81, 7.5], *P* = .11), and grade III or IV cerebral hemorrhages were observed in 18 of 114 (15.8%) and 11/114 (9.6%) infants in the ibuprofen and placebo groups, respectively (aRR 1.57, 95% CI 0.78-3.16, *P* = .20). No significant difference between treatment groups was observed for other adverse events such as renal failure, necrotizing enterocolitis, or systemic hypotension/circulatory shock (Table V).

Multiple Imputation and Per-Protocol Analysis

An intention-to-treat analysis that used multiple imputation for missing primary-outcome data showed results that were similar to those of the primary analysis (114 children in each group; aRR 0.99; 95% CI 0.85-1.15, *P* = .86) (Table VI; available at www.jpeds.com).

In the per-protocol analysis (Table VI), the ibuprofen group was constituted of 108 infants and the placebo group of 113 infants (the Figure provides an explanation of group numbers). Primary outcome was known for 102 and 101 infants, respectively. Survival without cerebral palsy occurred in 73 of 102 (71.6%) vs 72/101 (71.3%) infants in the ibuprofen and placebo groups, respectively. A per-protocol analysis that used multiple imputation for missing primary-outcome data showed results that were similar to those of the primary per-protocol analysis (Table VI).

Table IV. Cardiac ultrasound results at day 3 and 14, open-label rescue ibuprofen treatments, and surgical ligation of the ductus arteriosus in the large and small ductus groups

	Large ductus group			Small ductus group		
Echocardiography data and rescue treatments	Placebo n = 114	Ibuprofen n = 114	*P* value Ibuprofen vs Placebo*	n = 109	*P* value vs placebo†	*P* value vs ibuprofen‡
Echocardiography at d 3						
Alive at d 3, n (%)	112 (98.2)	112 (98.2)	-	107 (98.2)		
No. with data (%)	110 (98.2)	102 (91.1)	-	105 (98.1)		
Patterns of flow						
No. with data (%)	87 (77.7)	95 (84.8)		90 (84.1)		
Closed ductus arteriosus, n (%)	17 (19.5)	66 (69.5)	<.001	38 (42.2)	.01	.002
Closing pattern, n (%)	17 (19.5)	9 (9.5)		20 (22.2)		
Pulsatile pattern, n (%)	37 (42.5)	13 (13.7)		23 (25.6)		
Growing pattern, n (%)	13 (14.9)	5 (5.3)		8 (8.9)		
Pulmonary hypertension pattern, n (%)	3 (3.4)	2 (2.1)		1 (1.11)		
Left atrial/aortic root ratio						
No. with data (%)	100 (89.3)	77 (68.8)	-	89 (83)		
Median value (IQR)	1.57 (1.3,1.8)	1.40 (1.20,1.65)	.029	1.42 (1.20, 1.73)	.11	.72
Mean left pulmonary artery velocity						
No. with data (%)	89 (79.5)	73 (65.2)	-	72 (67.3)		
Median value (IQR), m/s	0.48 (0.39,0.59)	0.38 (0.32,0.45)	<.001	0.47 (0.37,0.59)	.89	.001
Telediastolic left pulmonary artery velocity						
No. with data (%)	90 (80.4)	63 (56.3)	-	72 (67.3)		
Median value (IQR), m/s	0.19 (0.12,0.27)	0.09 (0.06,0.14)	<.001	0.14 (0.08,0.30)	.18	.001
Echocardiography at d 14						
No. alive at d 14 (%)	106 (93.0)	98 (86.0)	-	100 (91.7)		
No. with data (%)	92 (91.7)	83 (84.7)	-	89 (89)		
Patterns of flow						
No. with data (%)	74 (69.8)	75 (76.6)	-	75 (75)		
Closed ductus arteriosus, n (%)	40 (54.0)	56 (74.7)	.49	48 (64.0)	.26	.21
Closing pattern, n (%)	11 (14.9)	3 (4.0)		6 (8.0)		
Pulsatile pattern, n (%)	18 (24.3)	12 (16.0)		20 (26.7)		
Growing pattern, n (%)	4 (5.4)	3 (4.0)		1 (1.3)		
Pulmonary hypertension pattern, n (%)	1 (1.3)	1 (1.3)		0 (0)		
Left atrial/aortic root ratio						
No. with data (%)	74 (69.8)	55 (56.1)	-	75 (75)		
Median value (IQR)	1.5 (1.3,1.8)	1.5 (1.3,1.7)	.27	1.5 (1.3,1.7)	.39	.67
Mean left pulmonary artery velocity						
No. with data (%)	64 (60.4)	45 (45.9)	-	68 (68)		
Median value (IQR), m/s	0.52 (0.39,0.69)	0.51 (0.37,0.66)	.65	0.50 (0.40,0.63)	.92	.71
Telediastolic left pulmonary artery velocity						
No. with data (%)	61 (57.5)	44 (44.9)	-	56 (56)		
Median value (IQR), m/s	0.13 (0.02,0.25)	0.10 (0.07,0.19)	.86	0.10 (0.06,0.18)	.38	.54
Open-label rescue treatment						
Open-label rescue ibuprofen treatment, n (%)	71 (62.3)	20 (17.5)	<.001	34 (31.2)	<.001	.02
Postnatal age of first rescue ibuprofen treatment (d), median (IQR)	4 (3,5)	4 (4,12)	.015	4 (3,8)	.53	.12
Open-label rescue started before d 3, n (%)	24 (33.8)	2 (10.0)	.049§	12 (35.3)	.78	.041
Open-label rescue started after d 3, n (%)	47 (66.2)	18 (90.0)		22 (64.7)		
Catecholamine and/or hydrocortisone hemisuccinate at time of rescue before d 3, n (%)	14/24 (58.3)	2/2 (100.0)	.24	10/12 (83.3)	.13	.53
Cumulative ibuprofen dose (mg/kg), median (IQR)	14.7 (0, 27.5)	20.0 (20.0, 20.0)	<.001	0.0 (0.0, 16.0)	<.001	<.001
Surgical ligation of ductus arteriosus, n (%)	15 (13.2)	8 (7.0)	.12	7 (6.4)	.09	.86
Median postnatal age at ductus ligation (IQR)	22 (14, 30)	28 (20,47)	.36	34 (26,48)	.12	.69

**P* values are expressed for ibuprofen vs placebo and were generated with the use of generalized estimating equation models adjusted for gestational age at birth and recruitment site and accounting for clustering of siblings from the same pregnancy.
†*P* values are expressed for small vs placebo and were generated with the use of generalized estimating equation models adjusted for gestational age at birth and recruitment site and accounting for clustering of siblings from the same pregnancy.
‡*P* values are expressed for small vs ibuprofen and were generated with the use of generalized estimating equation models adjusted for gestational age at birth and recruitment site and accounting for clustering of siblings from the same pregnancy.
§Fisher exact test.

Complementary Analyses

Two additional analyses were performed, the first on infants whose birth z score was less than −1. This analysis concerned only 29 enrolled infants in the large PDA group, (placebo = 11; ibuprofen = 18). There was no significant difference in the primary outcome between the groups: ibuprofen = 4/15 (26.7%); placebo = 4/11 (36.4%), aRR = 0.59 (0.17, 2.04). Nor did the primary outcome differ between the placebo and the small ductus group (Table III), aRR 0.95; 95% CI 0.59-1.51, *P* = .81. In the second complementary analysis evaluating ductus diameter measurements before 12 hours as a biomarker for identifying infants at risk for developing

a hemodynamically significant PDA, we observed a sensitivity of 0.68, 95% CI (0.58, 0.76), a specificity of 0.64, 95% CI (0.55,0.72), a positive likelihood ratio of 1.9, 95% CI (1.4, 2.4), and a negative likelihood ratio of 0.51, 95% CI (0.37, 0.69).

Discussion

In our trial, infants with a large PDA were treated with either ibuprofen or placebo within 12 hours of birth. Infants in both groups received open-label ibuprofen "rescue" treatment 3-4 days later if the large PDA persisted and met clinical and echocardiographic features consistent with a moderate-to-large hemodynamically significant PDA (Methods section). Although infants receiving early ibuprofen treatment had a lower incidence of hemodynamically significant PDA and decreased left-to-right pulmonary shunt at day 3 (as indicated by the decreased incidence of early pulmonary hemorrhage and decreased left pulmonary flow), this reduction was not associated with a reduction in mortality or morbidity at 36 weeks of postmenstrual age. Nor was there a difference in the rate of our primary outcome: survival without cerebral palsy at 2 years. These results are consistent with a previous exploratory study, performed in Australia, using indomethacin for early echocardiography-targeted treatment.[12] Our trial extends the findings from prior RCTs of prophylactic PDA treatment[6-8] by demonstrating that prophylactic treatment of infants who are at increased risk for developing a hemodynamically significant PDA does not appear to alter the rate of morbidities at 36 weeks or 2 years compared with a placebo approach that relied on back-up rescue therapy several days later.

We were surprised that the reduction in pulmonary hemorrhage before 3 days was not associated with a decrease in the rate of IVH and an improvement in later outcomes because pulmonary hemorrhage is a risk factor for high grade IVH[39] and its prevention might be expected to result in improved neurodevelopmental outcomes. Possible explanations for the lack of long-term benefits of our tested strategy include potential toxicities of ibuprofen that might counterbalance the potential benefits of reduced pulmonary hemorrhages, and the multifactorial etiologies that contribute to death and cerebral palsy . We found nonsignificant trends toward increased rates of high grade IVH and isolated gastrointestinal perforations in the ibuprofen group. These adverse events have previously been reported for ibuprofen, although not identified as more frequent in a meta-analysis of ibuprofen treatment trials.[11] Another meta-analysis[40] on presymptomatic-targeted treatment of PDA did not assess high grade IVH and isolated gastrointestinal perforation. Cerebral palsy is a multifactorial disease, and it may be that a single early intervention is unlikely to affect this outcome.[41]

We did find several secondary outcomes that differed between the 2 treatment approaches. Infants treated with ibuprofen had higher scores on several parts of the ASQ compared with placebo-treated infants. There were also several significant differences and trends between the 2 treatment approaches when compared with infants in the small ductus group. Infants in the placebo-treated large PDA group were ventilated for longer durations, had more pulmonary hemorrhages and cerebral palsy, and worse neurodevelopmental skills (fine motor skills, total ASQ score, and problem solving) compared with infants in the small ductus group. In contrast, when infants in the small ductus group were compared with those in the ibuprofen-treated large PDA group there were no differences in any of these outcomes (Table III). These findings regarding the potential consequences of a hemodynamically significant PDA shunt during the first days after birth on neurodevelopmental and respiratory outcomes warrant further investigations.

Our trial has several limitations. It cannot address the question of whether a large PDA should be closed or allowed to persist during the neonatal period because the high rate of early backup open-label PDA treatment at 3-4 days insured that by 14 days, 70% of the infants in both study groups had a closed or closing ductus flow pattern (Table IV). Although infants receiving open-label back-up treatment met prespecified echocardiographic criteria before being treated, the high rate of early PDA back-up treatment impedes definitive conclusions about an early treatment approach compared with a conservative approach where the PDA is allowed to persist for several weeks in spite of clinical and echocardiographic evidence of a large left-to-right shunt. The issue of early back-up treatment has been a limitation in many other double-blind randomized trials.[7,15,42] It is interesting to note that back-up treatment was not uncommon even among infants who had a small PDA within 12 hours of birth (who were not part of the randomized trial). Thirty-one percent met our rescue criteria and were treated with open-label ibuprofen at a median age of 4 days (Table IV).

As stated above, infants receiving open-label back-up treatment met prespecified echocardiographic criteria before being treated. However, our criteria for identifying PDA shunts qualifying for open-label treatment may have had significant interobserver variability and are surrogates, not perfect indicators, of the magnitude of ductus left-to-right shunts.[43] We used these imperfect criteria as they have been used in earlier trials and enable us to compare our results with those previously reported. Although several new scoring systems have been proposed to classify PDA shunt severity,[44,45] their accuracy and generalizability still require confirmation by investigators from other centers. Future trials will be needed to investigate more comprehensive early scoring systems.

The high rate of spontaneous and nonspontaneous ductus closure among the placebo-treated infants is another limitation of our study. Despite the use of early targeted echocardiography to identify infants with a large PDA, 39% of the PDAs in the placebo group had either closed or had a closing ductus flow pattern when examined 2 days later (Table IV). In addition to the difficulties and variability in accurately measuring ductal dimension, our results suggest that the early measurement of ductus diameter (at 12 hours after

ARTICLE IN PRESS

birth) is probably an insufficient biomarker to discriminate PDAs that will remain open with a large shunt from those that will spontaneously close in the next few days. Because of the high rate of early spontaneous ductus closure and the frequent use of early open-label rescue treatment in the placebo group, our estimate of ibuprofen's effect may be shifted toward the null hypothesis, meaning that the true effect of early ibuprofen treatment may be greater than what was observed in our study if the hemodynamically significant PDA in the placebo group had remained open for a much longer period of time.

Another limitation is that the power of the study was much lower than anticipated. When the study protocol was initially developed its hypothesis was built on the rates observed during the EPIPAGE I study (births in 1997). In our current study, the placebo control group had a survival without cerebral palsy rate of more than twice what had been expected from the EPIPAGE I study. This improved outcome is consistent with the results of the recently published EPIPAGE II study (from 2011 births) which also showed a significant increase over time in cerebral palsy -free survival compared with the earlier period.[46] Because of the wide CIs of our primary outcome, a 17% increase or decrease in survival without cerebral palsy was compatible with the results of our study.

In our study, based on measurements of PDA diameter alone, early targeted use of ibuprofen in premature infants between $24^{0/7}$and $27^{6/7}$ weeks of gestation did not change survival or rates of cerebral palsy compared with a placebo approach that relied on back-up rescue therapy several days later. Unlike the prior Australian trial which used a similar approach (but with indomethacin instead of ibuprofen), we did not observe a decrease in IVH frequency. Not all nonsteroidal antiinflammatory drugs have the same effect. Whereas indomethacin prophylaxis has previously been shown to decrease the risk of severe IVH and improve long-term outcomes among boys,[47] the same has yet to be shown for ibuprofen.

To close or not to close a large PDA during the first weeks remains an open question and will require greater equipoise on the part of investigators toward the need for back-up treatments. Observational studies[5,14] support early closure of PDA, but have never been confirmed by an interventional study using indomethacin or ibuprofen. Future RCTs should prioritize the identification of populations at greatest risk of both physiologic and clinical consequences from the shunt to reduce the number of infants being exposed to indomethacin or ibuprofen. The use of an investigational drug with fewer side effects, such as acetaminophen, might be another option if it proves to be an effective drug for closing large PDAs in infants with extreme prematurity.[48,49] Alternatively, an early transcatheter percutaneous closure approach might be able to provide definitive PDA closure if it can be performed safely in infants with extreme prematurity.[50,51] ■

We acknowledge the contributions of the late Professor Véronique Gournay, who was the principal investigator of this trial and conceptualized, designed the study, and obtained grant support from the French Health Ministry for the study. We thank Professor Nick Evans from the department of Newborn Care, Royal Prince Alfred Hospital and University of Sydney, Sydney, New South Wales, Australia, for sharing the Ductal Echocardiographic Targeting and Early Closure Trial protocol with Professor Gournay and helping her write the TRIOCAPI protocol. We thank all the parents who have agreed to their infants' participation in this clinical trial. We also thank all the clinical research staff and physicians in the 11 NICUs who made this clinical trial possible.

Submitted for publication Aug 23, 2020; last revision received Nov 30, 2020; accepted Dec 3, 2020.

Reprint requests: Jean-Christophe Rozé, MD, PhD, Neonatal Care Unit, Nantes University Hospital, 38 Boulevard Jean Monnet, 44000 Nantes, France. E-mail: jean-christophe.roze@inserm.fr

Data Statement

Data sharing statement available at www.jpeds.com.

References

1. Evans N. Preterm patent ductus arteriosus: a continuing conundrum for the neonatologist? Semin Fetal Neonatal Med 2015;20:272-7.
2. Benitz WE and Committee on Fetus and Newborn. Patent ductus arteriosus in preterm infants. Pediatrics 2016;137:e20153730.
3. Jensen EA, Dysart KC, Gantz MG, Carper B, Higgins RD, Keszler M, et al. Association between use of prophylactic indomethacin and the risk for bronchopulmonary dysplasia in extremely preterm infants. J Pediatr 2017;186:34-40.
4. Edstedt Bonamy AK, Gudmundsdottir A, Maier R, Toome L, Zeitlin J, Bonet M, et al. Patent ductus arteriosus treatment in very preterm infants: a European population-based cohort study (EPICE) on variation and outcomes. Neonatology 2017;111:367-75.
5. Rozé JC, Cambonie G, Marchand-Martin L, Gournay V, Durrmeyer X, Durox M, et al. Association between early screening for patent ductus arteriosus and in-hospital mortality among extremely preterm Infants. JAMA 2015;313:2441-8.
6. Schmidt B, Davis P, Moddemann D, Ohlsson A, Roberts RS, Saigal S, et al. Long-term effects of indomethacin prophylaxis in extremely-low-birth-weight infants. N Engl J Med 2001;344:1966-72.
7. Gournay V, Roze JC, Kuster A, Daoud P, Cambonie G, Hascoet JM, et al. Prophylactic ibuprofen versus placebo in very premature infants: a randomised, double-blind, placebo-controlled trial. Lancet 2004;364:1939-44.
8. Van Overmeire B, Allegaert K, Casaer A, Debauche C, Decaluwé W, Jespers A, et al. Prophylactic ibuprofen in premature infants: a multicentre, randomised, double-blind, placebo-controlled trial. Lancet 2004;364:1945-9.
9. Gournay V, Savagner C, Thiriez G, Kuster A, Rozé JC. Pulmonary hypertension after ibuprofen prophylaxis in very preterm infants. Lancet 2002;359:1486-8.
10. Fowlie PW, Davis PG, McGuire W. Prophylactic intravenous indomethacin for preventing mortality and morbidity in preterm infants. Cochrane Database Syst Rev 2010;7:CD000174.
11. Ohlsson A, Shah SS. Ibuprofen for the prevention of patent ductus arteriosus in preterm and/or low birth weight infants. Cochrane Database Syst Rev 2020;1:CD004213.
12. Cooke L, Steer P, Woodgate P. Indomethacin for asymptomatic patent ductus arteriosus in preterm infants. Cochrane Database Syst Rev 2003;2:CD003745.
13. Sosenko IR, Fajardo MF, Claure N, Bancalari E. Timing of patent ductus arteriosus treatment and respiratory outcome in premature infants: a double-blind randomized controlled trial. J Pediatr 2012;160:929-35.e1.
14. Liebowitz M, Clyman RI. prophylactic indomethacin compared with delayed conservative management of the patent ductus arteriosus in

extremely preterm infants: effects on neonatal outcomes. J Pediatr 2017;187:119-26.e1.
15. Schena F, Francescato G, Cappelleri A, Picciolli I, Mayer A, Mosca F, et al. Association between hemodynamically significant patent ductus arteriosus and bronchopulmonary dysplasia. J Pediatr 2015;166:1488-92.
16. Sellmer A, Bjerre JV, Schmidt MR, McNamara PJ, Hjortdal VE, Host B, et al. Morbidity and mortality in preterm neonates with patent ductus arteriosus on day 3. Arch Dis Child Fetal Neonatal Ed 2013;98:F505-10.
17. Clyman RI, Hills NK, Liebowitz M, Johng S. relationship between duration of infant exposure to a moderate-to-large patent ductus arteriosus shunt and the risk of developing bronchopulmonary dysplasia or death before 36 weeks. Am J Perinatol 2020;37:216-23.
18. Kluckow M, Jeffery M, Gill A, Evans N. A randomised placebo-controlled trial of early treatment of the patent ductus arteriosus. Arch Dis Child Fetal Neonatal Ed 2014;99:F99-104.
19. Papile LA, Burstein J, Burstein R, Koffler H. Incidence and evolution of subependymal and intraventricular hemorrhage: a study of infants with birth weights less than 1,500 gm. J Pediatr 1978;92:529-34.
20. Evans N, Gournay V, Cabanas F, Kluckow M, Leone T, Groves A, et al. Point-of-care ultrasound in the neonatal intensive care unit: international perspectives. Semin Fetal Neonatal Med 2011;16:61-8.
21. Kluckow M, Evans N. Early echocardiographic prediction of symptomatic patent ductus arteriosus in preterm infants undergoing mechanical ventilation. J Pediatr 1995;127:774-9.
22. Kluckow M, Paradisis M, Anjiti R, Gill A, Osborne D, Evans N. Duct diameter is determined more by postnatal age than gestational age. J Paediatr Child Health 2008;44:A23.
23. El Hajjar M, Vaksmann G, Rakza T, Kongolo G, Storme L. Severity of the ductal shunt: a comparison of different markers. Arch Dis Child Fetal Neonatal Ed 2005;90:F419-22.
24. McNamara PJ, Sehgal A. Towards rational management of the patent ductus arteriosus: the need for disease staging [published correction appears in Arch Dis Child Fetal Neonatal Ed 2008;93:F78. Arch Dis Child Fetal Neonatal Ed 2007;92:F424-7.
25. Hirt D, Van Overmeire B, Treluyer JM, Langhendries JP, Marguglio A, Eisinger MJ, et al. An optimized ibuprofen dosing scheme for preterm neonates with patent ductus arteriosus, based on a population pharmacokinetic and pharmacodynamic study. Br J Clin Pharmacol 2008;65:629-36.
26. Amiel-Tison C, Stewart A. Follow up studies during the first five years of life: a pervasive assessment of neurological function. Arch Dis Child 1989;64:496-502.
27. Hanf M, Nusinovici S, Rouger V, Olivier M, Berlie I, Flamant C, et al. Cohort Profile: Longitudinal study of preterm infants in the Pays de la Loire region of France (LIFT cohort). Int J Epidemiol 2017;46:1396-1397h.
28. Gosselin J, Amiel-Tison C, Infante-Rivard C, Fouron C, Fouron JC. Minor neurological signs and developmental performance in high risk children at preschool age. Dev Med Child Neurol 2002;44:323-8.
29. Palisano R, Rosenbaum P, Walter S, Russell D, Wood E, Galuppi B. Development and reliability of a system to classify gross motor function in children with cerebral palsy. Dev Med Child Neurol 1997;39:214-23.
30. Walsh MC, Wilson-Costello D, Zadell A, Newman N, Fanaroff A. Safety, reliability, and validity of a physiologic definition of bronchopulmonary dysplasia. J Perinatol 2003;23:451-6.
31. Walsh MC, Kliegman RM. Necrotizing enterocolitis: treatment based on staging criteria. Pediatr Clin North Am 1986;33:179-201.
32. Volpe JJ. Neurobiology of periventricular leukomalacia in the premature infant. Pediatr Res 2001;50:553-62.
33. An international classification of retinopathy of prematurity. II. The classification of retinal detachment. The International Committee for the Classification of the Late Stages of Retinopathy of Prematurity. Arch Ophthalmol 1987;105:906-12.
34. Squires J, Bricker D, Potter L. Revision of a parent-completed development screening tool: Ages and Stages Questionnaires. J Pediatr Psychol 1997;22:313-28.
35. Frondas-Chauty A, Simon L, Branger B, Gascoin G, Flamant C, Ancel PY, et al. Early growth and neurodevelopmental outcome in very preterm infants: impact of gender. Arch Dis Child Fetal Neonatal Ed 2014;99:F366-72.
36. Flamant C, Branger B, Nguyen The Tich S, de la Rochebrochard E, Savagner C, Berlie I, et al. Parent-completed developmental screening in premature children: a valid tool for follow-up programs. PLoS One 2011;6:e20004.
37. Ancel PY, Livinec F, Larroque B, Marret S, Arnaud C, Pierrat V, et al. Cerebral palsy among very preterm children in relation to gestational age and neonatal ultrasound abnormalities: the EPIPAGE cohort study. Pediatrics 2006;117:828-35.
38. Schulz KF, Altman DG, Moher D , CONSORT Group. CONSORT 2010 Statement: updated guidelines for reporting parallel group randomised trials. Trials 2010;11:32.
39. Pandit PB, O'Brien K, Asztalos E, Colucci E, Dunn MS. Outcome following pulmonary haemorrhage in very low birthweight neonates treated with surfactant. Arch Dis Child Fetal Neonatal Ed 1999;81:F40-4.
40. Farooqui MA, Elsayed YN, Jeyaraman MM, Dingwall O, Tagin M, Zarychanski R, et al. Pre-symptomatic targeted treatment of patent ductus arteriosus in preterm newborns: a systematic review and meta-analysis. J Neonatal Perinatal Med 2019;12:1-7.
41. Marlow N, Doyle LW, Anderson P, Johnson S, Bhatt-Mehta V, Natalucci G, et al. Assessment of long-term neurodevelopmental outcome following trials of medicinal products in newborn infants. Pediatr Res 2019;86:567-72.
42. Jones LJ, Craven PD, Attia J, Thakkinstian A, Wright I. Network meta-analysis of indomethacin versus ibuprofen versus placebo for PDA in preterm infants. Arch Dis Child Fetal Neonatal Ed 2011;96:F45-52.
43. Broadhouse KM, Price AN, Durighel G, Cox DJ, Finnemore AE, Edwards AD, et al. Assessment of PDA shunt and systemic blood flow in newborns using cardiac MRI. NMR Biomed 2013;26:1135-41.
44. El-Khuffash A, Levy PT, Gorenflo M, Frantz ID III. The definition of a hemodynamically significant ductus arteriosus. Pediatr Res 2019;85:740-1.
45. Fink D, El-Khuffash A, McNamara PJ, Nitzan I, Hammerman C. Tale of two patent ductus arteriosus severity scores: similarities and differences. Am J Perinatol 2018;35:55-8.
46. Pierrat V, Marchand-Martin L, Arnaud C, Kaminski M, Resche-Rigon M, Lebeaux C, et al., EPIPAGE-2 Writing Group. Neurodevelopmental outcome at 2 years for preterm children born at 22 to 34 weeks' gestation in France in 2011: EPIPAGE-2 cohort study. BMJ 2017;358:j3448.
47. Ment LR, Vohr BR, Makuch RW, Westerveld M, Katz KH, Schneider KC, et al. Prevention of intraventricular hemorrhage by indomethacin in male preterm infants. J Pediatr 2004;145:832-4.
48. Härkin P, Härmä A, Aikio O, Valkama M, Leskinen M, Saarela T, et al. Paracetamol accelerates closure of the ductus arteriosus after premature birth: a randomized trial. J Pediatr 2016;177:72-7.e2.
49. Ohlsson A, Shah PS. Paracetamol (acetaminophen) for patent ductus arteriosus in preterm or low birth weight infants. Cochrane Database Syst Rev 2018;4:CD010061.
50. Sathanandam S, Agrawal H, Chilakala S, Johnson J, Allen K, Knott-Craig C, et al. Can transcatheter PDA closure be performed in neonates ≤1000 grams? The Memphis experience. Congenit Heart Dis 2019;14:79-84.
51. Regan W, Benbrik N, Sharma SR, Auriau J, Bouvaist H, Bautista-Rodriguez C, et al. Improved ventilation in premature babies after transcatheter versus surgical closure of patent ductus arteriosus. Int J Cardiol 2020;311:22-7.

Table I. Preplanned secondary outcomes evaluated as part of the TRIOCAPI Trial and adverse events

Preplanned secondary outcomes
Open-label rescue treatment
Surgical ligation
Duration of mechanical ventilation, noninvasive ventilation, and oxygen delivery
ASQ completed at 24 mo of corrected age
Death and survival without severe morbidity at 36 wk of corrected age or discharge, whichever came first
Severe morbidities
Bronchopulmonary dysplasia*
Necrotizing enterocolitis†
IVH grade III-IV‡
Periventricular leukomalacia§
Others morbidities or adverse events
Retinopathy of prematurity (≥stage 2)¶
Gastrointestinal bleeding, isolated gastrointestinal perforation
Thrombocytopenia (platelet count <50 000/mm^3)
Severe pulmonary hemorrhage**
Pulmonary hypertension
Renal failure††
Late-onset sepsis‡‡
Severe hypotension§§
Multiple organ failure

*Bronchopulmonary dysplasia, defined by the use of supplemental oxygen at 36 weeks of postmenstrual age, using Walsh's room air challenge test.[30]
†Necrotizing enterocolitis, defined as a modified Bell stage 2b or more.[31]
‡IVH grade III-IV.[19]
§Periventricular leukomalacia.[32]
¶Retinopathy of prematurity (≥stage 2).[33]
**Pulmonary hemorrhage, defined as the combination of 2 consecutive bloody tracheal aspirates, an increase in $FiO_2 \geq 0\%$ and/or an increase in mean airway pressure ≥2 cm H_2O.
††Renal failure, defined by creatinine >150 μmol/L or oliguria less than 0.5/mL/kg/hour.
‡‡Late-onset sepsis requiring antibiotic therapy for more than 7 days.
§§Severe hypotension, defined as mean arterial blood pressure (in mm Hg) below gestational age (in weeks) for ≥4 hours despite maximal inotropic drug doses according to local practices.

Table V. Adverse events during hospital stay before 37 weeks of postmenstrual age

Adverse events	Large ductus group: Placebo n = 114	Large ductus group: Ibuprofen n = 114	aRRs [95% CI]*	*P* value Ibuprofen vs placebo	Small ductus group n = 109	*P* value Small ductus vs ibuprofen	*P* value Small ductus vs placebo
At least 1 adverse event, n (%)	93 (81.6)	102 (89.5)	1.02 [0.94-1.11]	.68	96 (88.0)	Not estimated	Not estimated
Mean number of events per infant, (SD)	2.5 (1.0)	2.4 (1.8)	-	.65	2.3 (1.7)	.99	.67
Death, n (%)	16 (14.0)	23 (20.2)	1.43 [0.80-2.53]	.22	19 (17.4)	.89	.30
Thrombocytopenia, n (%)	7 (6.1)	3 (2.6)	0.43 [0.11-1.62]	.21	8 (7.3)	.66	.96
Pulmonary hemorrhage during the first 3 d after birth, n (%)	9 (7.9)	2 (1.8)	0.22 [0.05-1.00]	.05	3 (2.8)	.62	.10
Pulmonary hemorrhage at any age, n (%)	13 (11.4)	5 (4.4)	0.38 [0.14-1.04]	.06	5 (4.6)	.94	.07
Pulmonary hypertension, n (%)	5 (4.4)	4 (3.5)	0.80 [0.22-2.89]	.74	1 (0.9)	.27	.19
Bronchopulmonary dysplasia at 36 wk of postmenstrual age,† n (%)	47 (41.2)	40 (35.1)	0.84 [0.61-1.18]	.32	37 (33.9)	.95	.29
Renal failure, n (%)	16 (14.0)	14 (12.3)	0.84 [0.43-1.63]	.60	11 (10.1)	.83	.45
Isolated gastrointestinal perforation, n (%)	4 (3.5)	10 (8.8)	2.46 [0.81-7.48]	.11	0	Not estimated	Not estimated
Necrotizing enterocolitis,‡ n (%)	6 (5.3)	5 (4.4)	0.83 [0.26-2.65]	.76	9 (8.3)	.22	.35
Late-onset sepsis, n (%)	79 (69.3)	75 (65.8)	0.94 [0.79-1.11]	.46	86 (78.9)	.01	.06
Severe sepsis,§ n (%)	4 (3.5)	7 (6.1)	1.75 [0.52-5.83]	.36	8 (7.3)	.70	.21
All circulatory events (hypotension and/or shock),¶ n (%)	56 (49.1)	55 (48.2)	0.90 [0.71-1.14]	.40	42 (38.5)	.86	.44
Multiple organ failure	0 (0.9)	3 (2.6)	Not estimated	-	4 (3.7)	Not estimated	Not estimated
Grade III or IV cerebral hemorrhage,** n (%)	11 (9.6)	18 (15.8)	1.57 [0.78-3.16]	.20	10 (9.2)	.27	.94
Periventricular leukomalacia, n (%)	3 (2.6)	5 (4.4)	1.69 [0.41-6.96]	.46	3 (2.8)	.45	.99
Retinopathy of prematurity stage >2,†† n (%)	5 (4.4)	3 (2.6)	0.59 [0.14-2.42]	.46	4 (3.7)	.59	.85

*Relative risks are expressed for ibuprofen vs placebo and were generated with the use of generalized estimating equation models adjusted for gestational age at birth and recruitment site and accounting for clustering of siblings from the same pregnancy.
†Use of supplemental oxygen at 36 weeks of postmenstrual age, using the Walsh room air challenge test.[22]
‡Stage 2b or more using the modified Bell classification.[23]
§All sepsis events requiring volume expansion or inotropic treatment.
¶All circulatory events requiring volume expansion or inotropic treatment.
**Serious intraventricular hemorrhages, defined as grades 3 or 4 intraventricular hemorrhage (using the four-level grading system).[19]
††According to international classification.[33]

Table VI. Survival without cerebral palsy at 24 months of corrected age in the large ductus group using the primary and sensitivity analysis

Models used	Placebo	Ibuprofen	aRR [95% CI]*	*P* value
Modified intention to treat analysis (cases with primary outcome),† n/N (%)	73/102 (71.6)	77/108 (71.3)	0.98 [0.83-1.16]	.84
Modified intention to treat analysis with multiple imputations),† n/N (%)	83/114 (72.6)	83/114 (72.6)	0.99 [0.85-1.15]	.86
Per protocol analysis, (cases with primary outcome),† n/N (%)	72/101 (71.3)	73/102 (71.6)	1.00 [0.84-1.18]	.96
Per protocol analysis with multiple imputations),† n/N (%)	81/113 (72.0)	79/108 (72.9)	1.00 [0.85-1.17]	.98

*Relative risks are expressed for ibuprofen vs placebo and were generated with the use of generalized estimating equation models adjusted for gestational age at birth and recruitment site and accounting for clustering of siblings from the same pregnancy.
†Two analyses were performed: an "intention to treat" analysis and a "per-protocol" analysis. Each analysis was performed with and without multiple imputations to account for infants who did not have a primary outcome recorded (**Figure**). In the intention-to-treat analysis, all enrolled infants were included in the analysis: placebo large ductus group (n = 114), ibuprofen large ductus group (n = 114). In the per protocol analysis, the numbers included were placebo large ductus group (n = 113, the infant randomized by error was withdrawn), ibuprofen large ductus group (n = 108, the 3 infants randomized by error and the 3 infants receiving only 1 dose of ibuprofen by error were withdrawn).
