# Supplementary material for: Patent ductus arteriosus, tracheal ventilation, and the risk of bronchopulmonary dysplasia
Source: Pediatr Res. 2021 Mar 31;91(3):652–8. doi: 10.1038/s41390-021-01475-w (PMC8904244; doi:10.1038/s41390-021-01475-w)
Supplement: Supplementary file 2 — Supplementary information [file 41390_2021_1475_MOESM2_ESM.docx]

Section Editor: 1

Comments to the Author:

A few minor issues still to be considered.  See also reviewer 1's suggested modification to the title.

The Editor wrote: “A few minor issues still to be considered.” Unfortunately, we did not see any “minor issues” listed under the Editor’s comment. Are there any? We have responded to Reviewer 1’s suggested modification (see below).

Reviewer: 1

Comments to the Author

Thanks for the opportunity to review the revised manuscript submitted by Clyman and colleagues.

I would like to thank the authors for providing detailed responses to my previous comments. Most of my queries have thus been addressed. Only a couple of minor comments remain:

1. Abstract: The authors did not clarify in their responses if this analysis was a post-hoc analysis. If yes, this should be clearly mentioned in the study design in the abstract and methods. As I have mentioned before, there is always a risk of introducing bias with any post-hoc exploratory analysis and this should be clearly communicated to the readers.

The reviewer asks whether our analysis was a post-hoc analysis or whether the variables and outcomes in our study for ‘prolonged PDA exposure’, ‘prolonged tracheal intubation’ and ‘BPD’ were pre-specified and pre-defined prior to the anlaysis. We thought we had addressed this question when we last responded to it after the manuscript’s first review. At that time we stated: “*Both the variables (‘prolonged PDA exposure’, ‘prolonged tracheal intubation’) and the outcome (BPD) were predefined. Our study was designed to validate and confirm the findings of our prior studies that explored the same hypothesis.*” Let me be clear: both our planned analyses for the current study and all the variables that were used in the current study were predefined prior to obtaining the data for the study. The purpose of the study was to validate the findings of two prior studies. Therefore, as a validation study, we planned, a priori, to use the exact same variables and outcome definitions that were used in the prior studies that we wanted to validate. By definition, a post-hoc study performs an analysis that was not specified before the data were seen. That is clearly NOT the case in our current study. The hypothesis and variables for this study were prespecified and defined before we even had the data to analyze.

To make this clear in the Abstract we have changed the wording of the “Study Design” to: “***Predefined definitions*** *of prolonged ventilation (≥10 days), hsPDA (≥14 days) and BPD (room air challenge test at 36 weeks) were used to analyze deidentified data from the multicenter TRIOCAPI RCT in a secondary analysis of the trial*.”

We have also added two new sentences in the beginning of the Methods section to clarify that the definitions of the variables and outcomes were predefined prior to the study analyses:

“*The goal of our study was to validate the findings of the two prior studies (*[*18*](#_ENREF_18)*,* [*19*](#_ENREF_19)*) that found an interaction between the duration of tracheal ventilation and the duration of PDA exposure on the incidence of BPD. As a validation study****, we used the same definitions for our study variables and outcomes that were used in the prior studies*** *(see below for definitions).*”

2. Difference in the definition of prolonged PDA exposure in this study compared to the PDA-TOLERATE secondary analysis: Thank you for providing a detailed rationale. Given that all secondary papers related to the PDA-TOLERATE trial have been widely read, this difference will be easily picked up by readers. I would suggest briefly discussing the PDA-TOLERATE secondary analysis and the reason for difference in the PDA exposure duration between the studies in 1-2 sentences. This might be helpful for researchers developing a future trial on early selective treatment of moderate-large PDAs.

We appreciate the Reviewer’s comments and have expanded the Statistical analysis section of the Methods section - discussing the difference in the definition for prolonged PDA exposure between the PDA-TOLERATE secondary analysis and our current study:

“*Prior observational studies have reported that infants <28 weeks’ gestation, who were exposed to a moderate-to-large PDA for longer than 7-14 days, had a significantly higher incidence of BPD than those exposed to shorter durations (the incidence of any-grade BPD (defined by the room air challenge test) appeared to be increased with PDA exposures ≥14 days (*[*16*](#_ENREF_16)*,* [*18*](#_ENREF_18)*,* [*19*](#_ENREF_19)*), while the incidence of more severe grades of BPD, grades 2 and 3 (*[*26*](#_ENREF_26)*), increased with somewhat shorter PDA exposures (≥7 days) (*[*16*](#_ENREF_16)*,* [*18*](#_ENREF_18)*,* [*19*](#_ENREF_19)*). …In the previously reported secondary analysis of the PDA-TOLERATE trial, that examined the interaction between duration of tracheal ventilation and duration of PDA exposure on the incidence of both BPD-any grade and BPD (grades 2 & 3), a duration of PDA exposure midway between ≥7 and ≥14 days (≥11 days) was used to define ‘prolonged PDA exposure’ (*[*16*](#_ENREF_16)*,* [*18*](#_ENREF_18)*,* [*19*](#_ENREF_19)*). However, in our current secondary analysis of the TRIOCAPI trial we used a duration of PDA exposure ≥14 days to define ‘prolonged PDA exposure’ since we only planned to examine the outcome BPD-any grade. (We were not able to examine the outcome BPD (grades 2 & 3) since the clinical practice among French neonatologists was to continue CPAP as the main respiratory support (rather than nasal cannula) late in the hospitalization, making it impossible to use the grading system defined by Jensen et al (*[*26*](#_ENREF_26)*)).”*

Title could be improved.

"Patent ductus arteriosus, tracheal ventilation, and the risk of bronchopulmonary dysplasia: a TRIOCAPI-trial post-hoc secondary analysis"

We disagree with the Reviewer’s proposed change in title. As stated above our study was NOT a post-hoc analysis and therefore should not be labeled as such. The title is left unchanged
